# Supplementary material for: The kinase domain of RIPK3 tunes its scaffolding functions
Source: Cell Death Differ. 2026 Feb 3;33(8):1523–35. doi: 10.1038/s41418-026-01677-x (PMC13434183; doi:10.1038/s41418-026-01677-x)

For Fig 1C

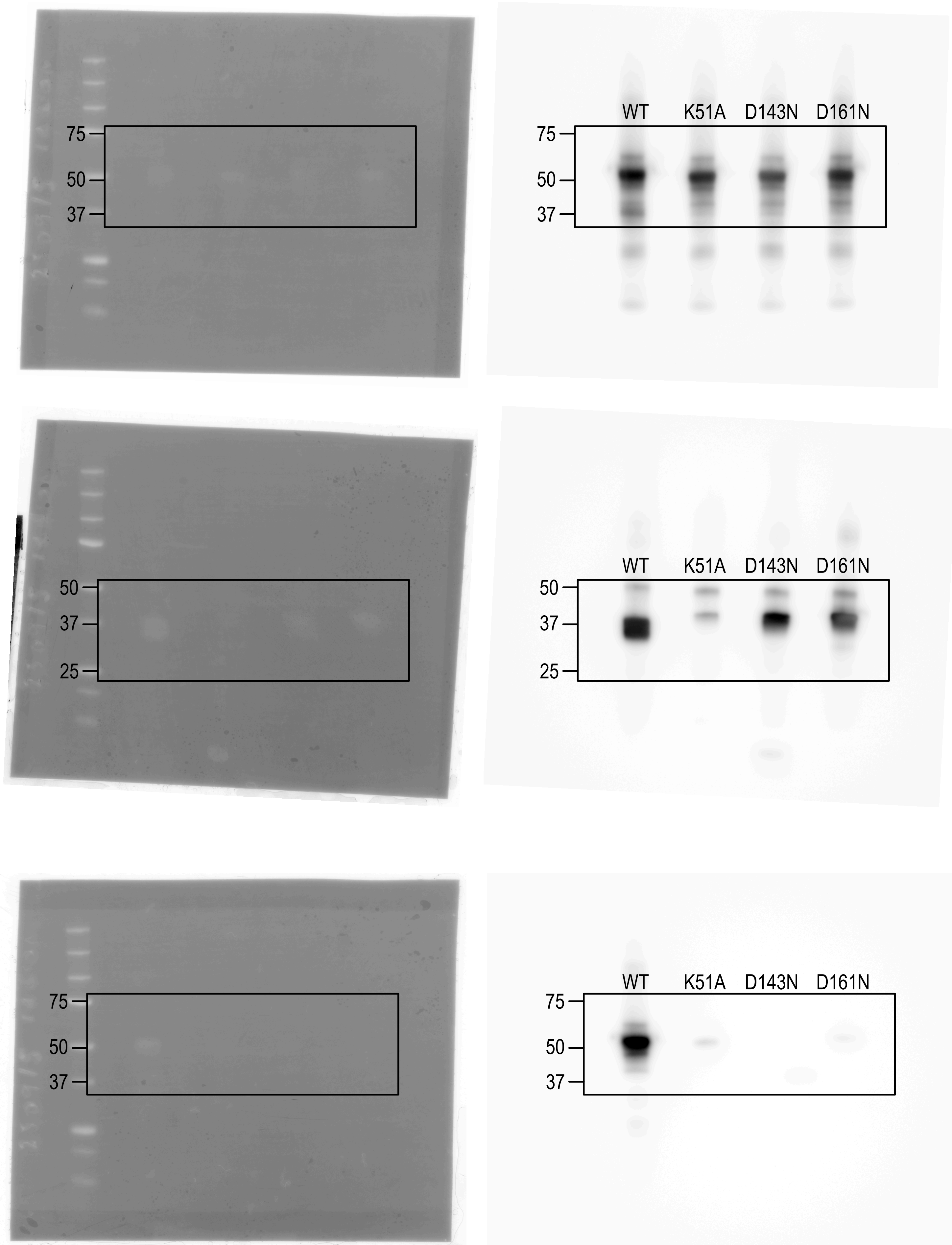
75
50
37
WT
K51A
D143N
D161N
75
50
37
3H1
50
37
25
WT
K51A
D143N
D161N
50
37
25
8G7
75
50
37
WT
K51A
D143N
D161N
75
50
37
pS345 (CST; D6E3G)

For Fig 1E

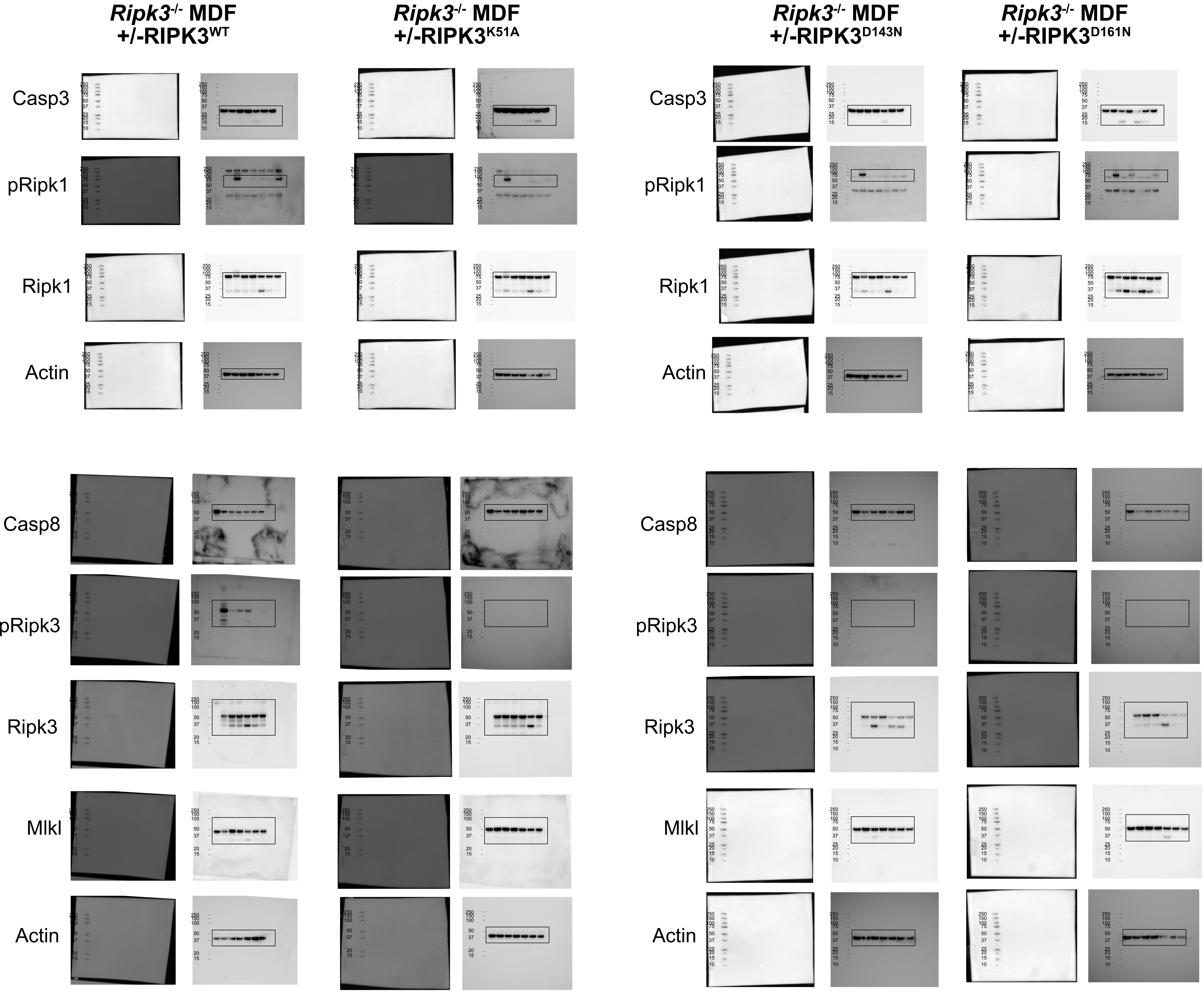

Ripk3-/- MDF +/-RIPK3WT
Ripk3-/- MDF +/-RIPK3K51A
Ripk3-/- MDF +/-RIPK3D143N
Ripk3-/- MDF +/-RIPK3D161N
Casp3
pRipk1
Ripk1
Actin
Casp8
pRipk3
Ripk3
Mlkl
Actin

For Fig 2A

Uncropped cellular thermal stability assay blots for anti-RIPK3

Uncropped cellular thermal stability assay blots for anti-MLKL

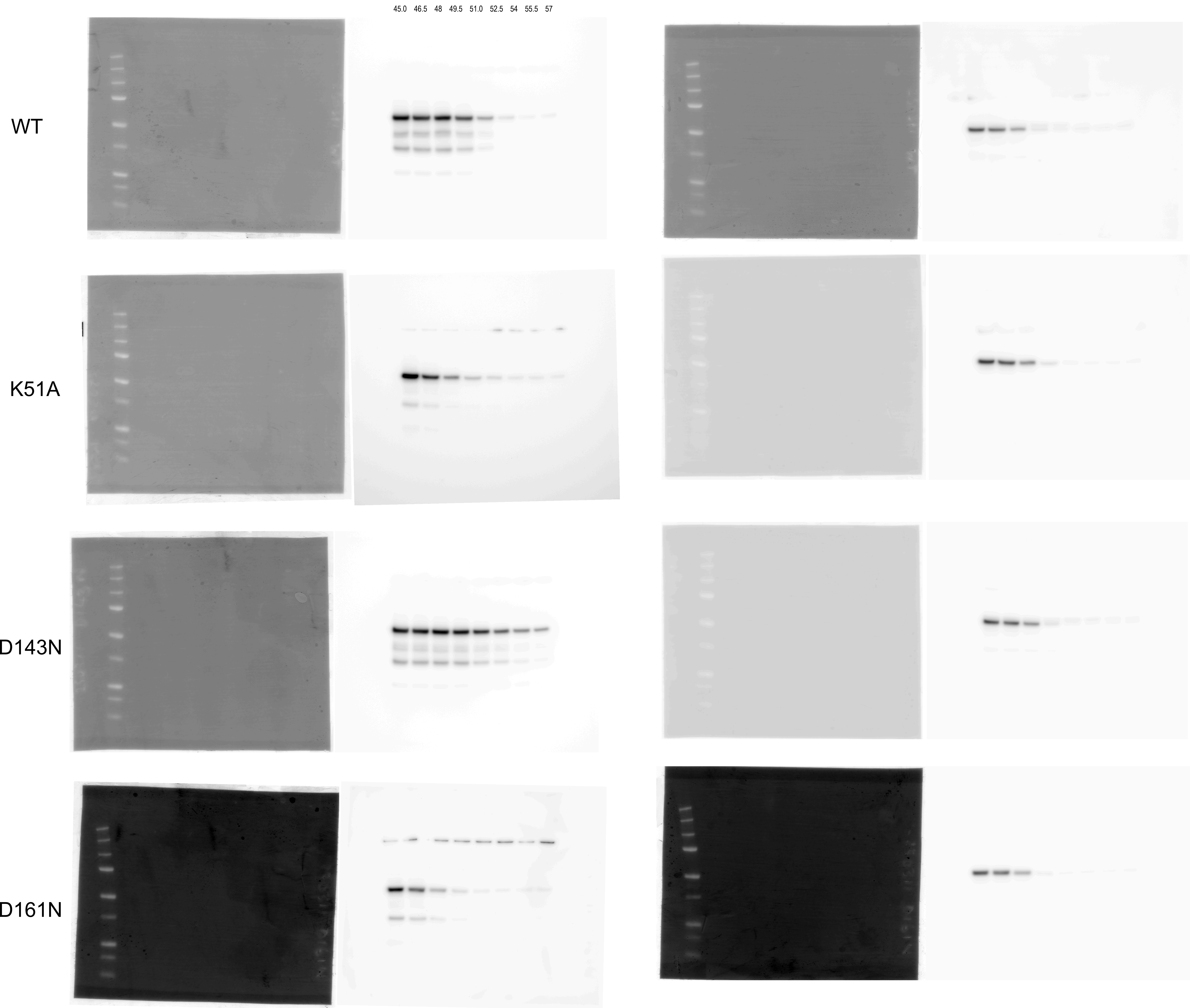

For Fig 2C

Uncropped FLAG-IP blots

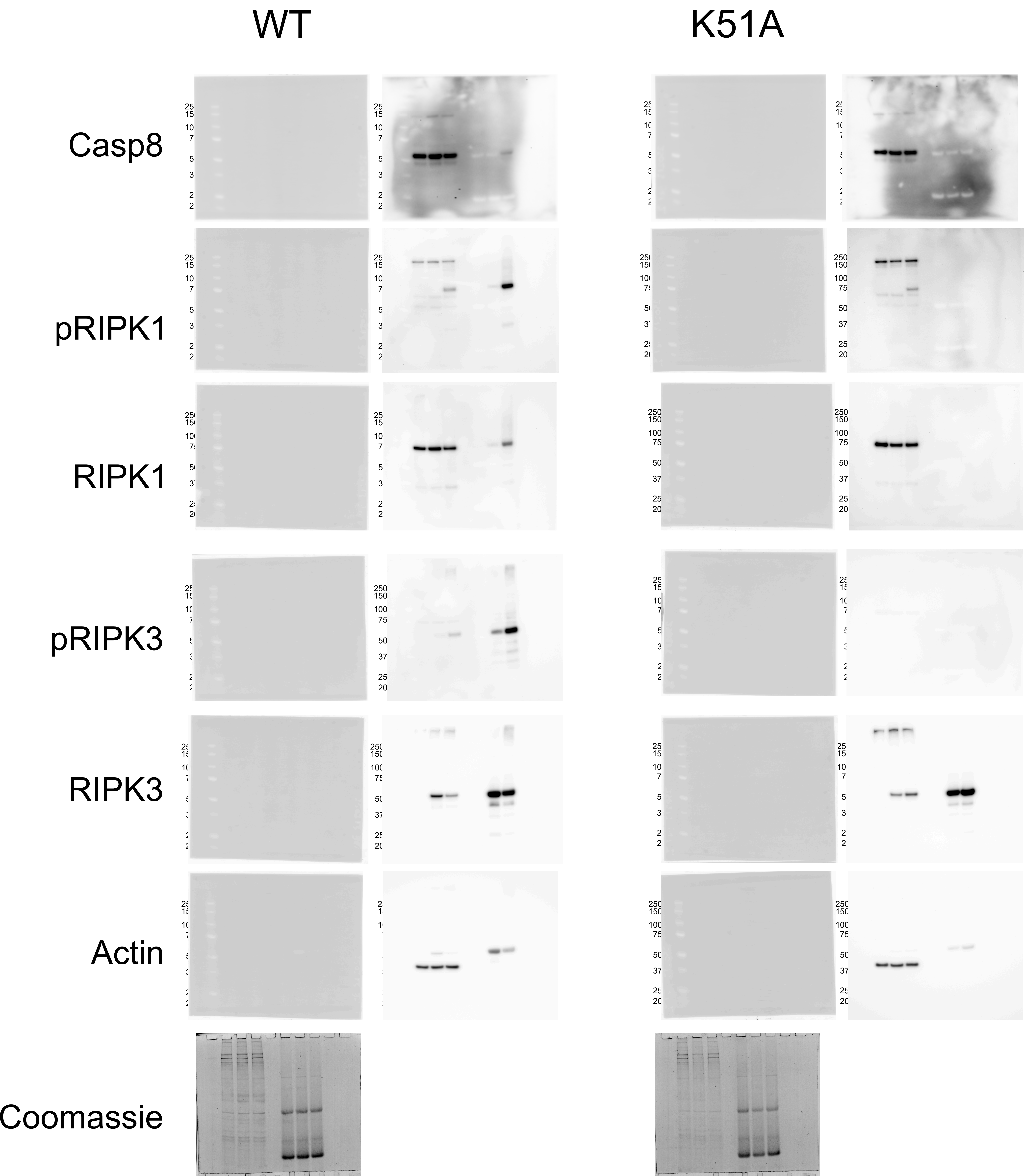

Uncropped FLAG-IP blots

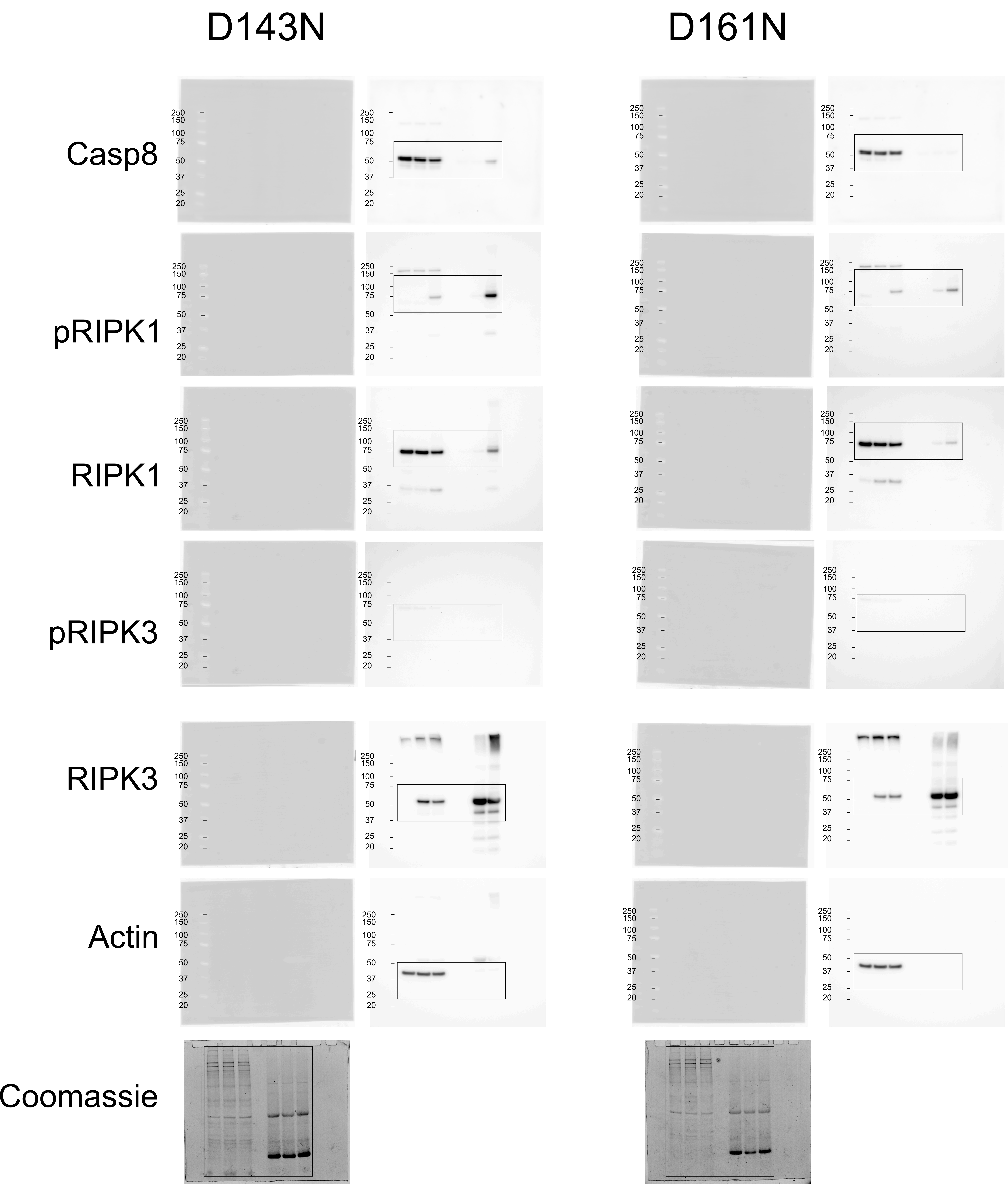

For Fig 3D

RIPK3

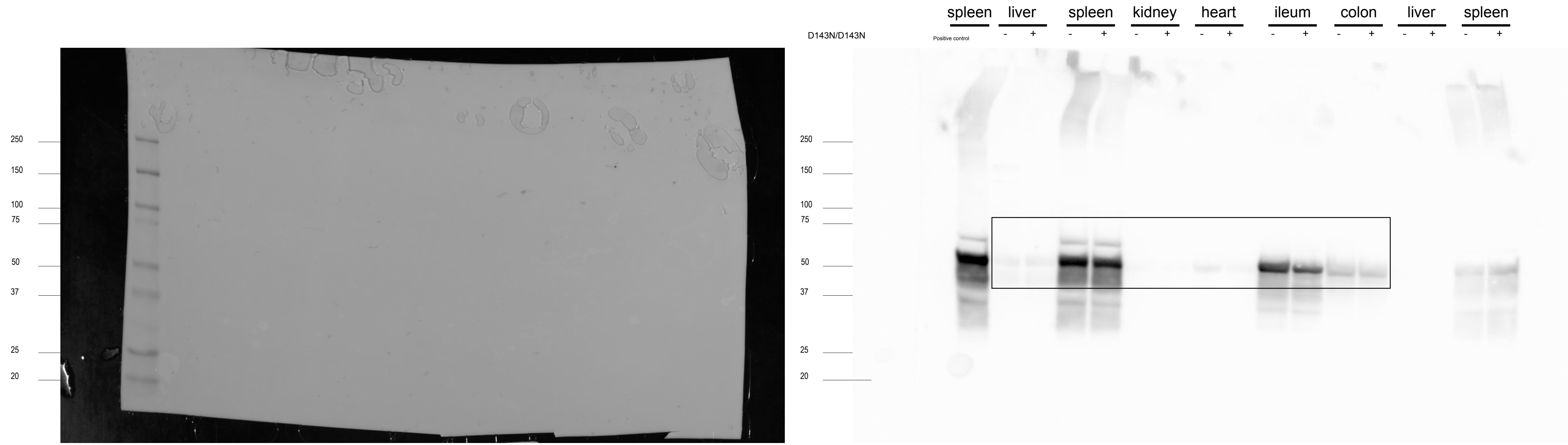

GAPDH

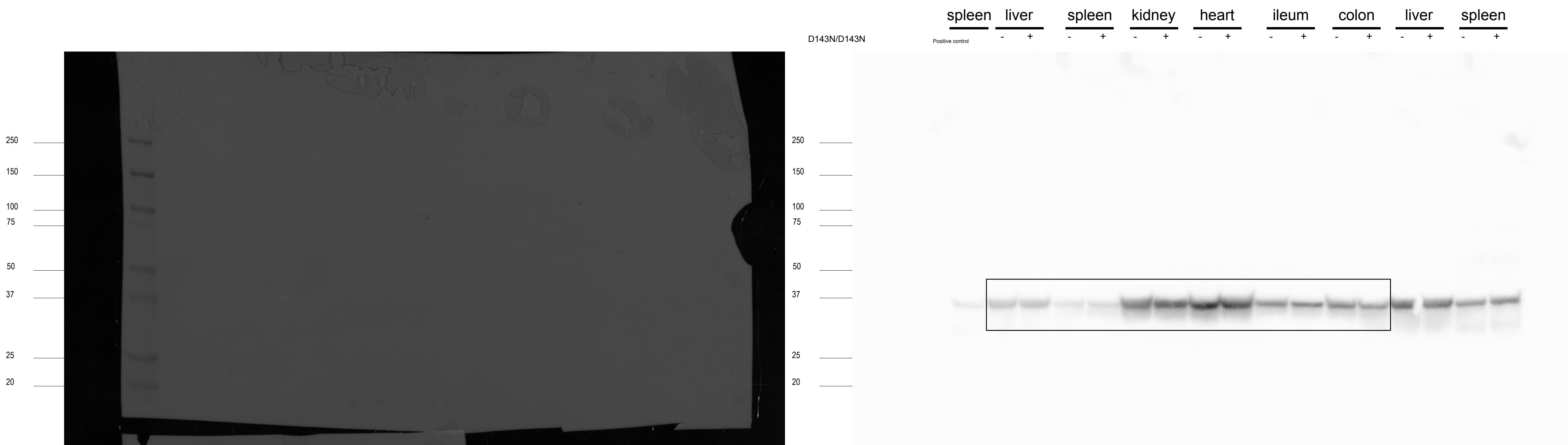

For Supplementary Fig 1C

RIPK3
53.2°C

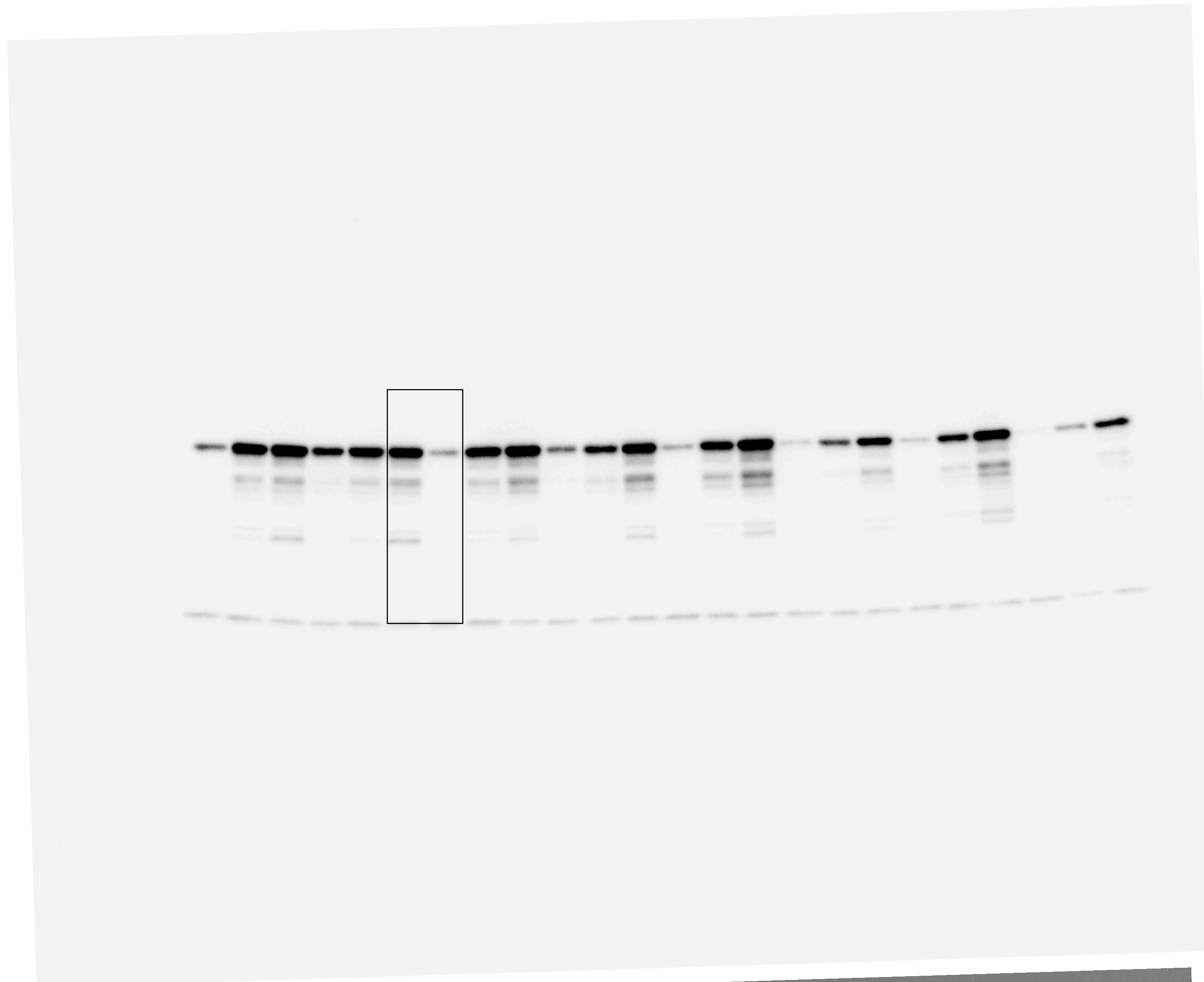

RIPK3
55.0°C

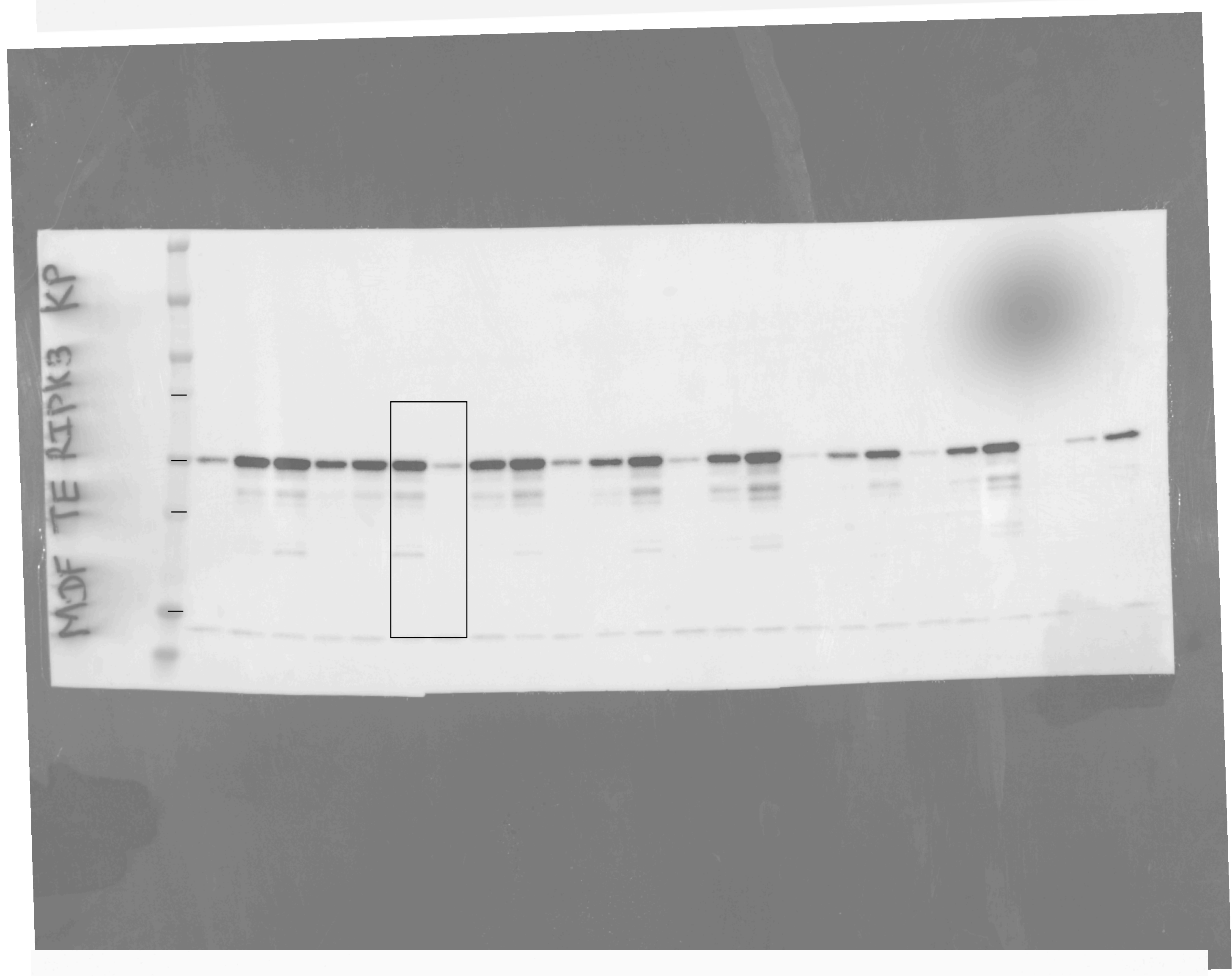

GAPDH

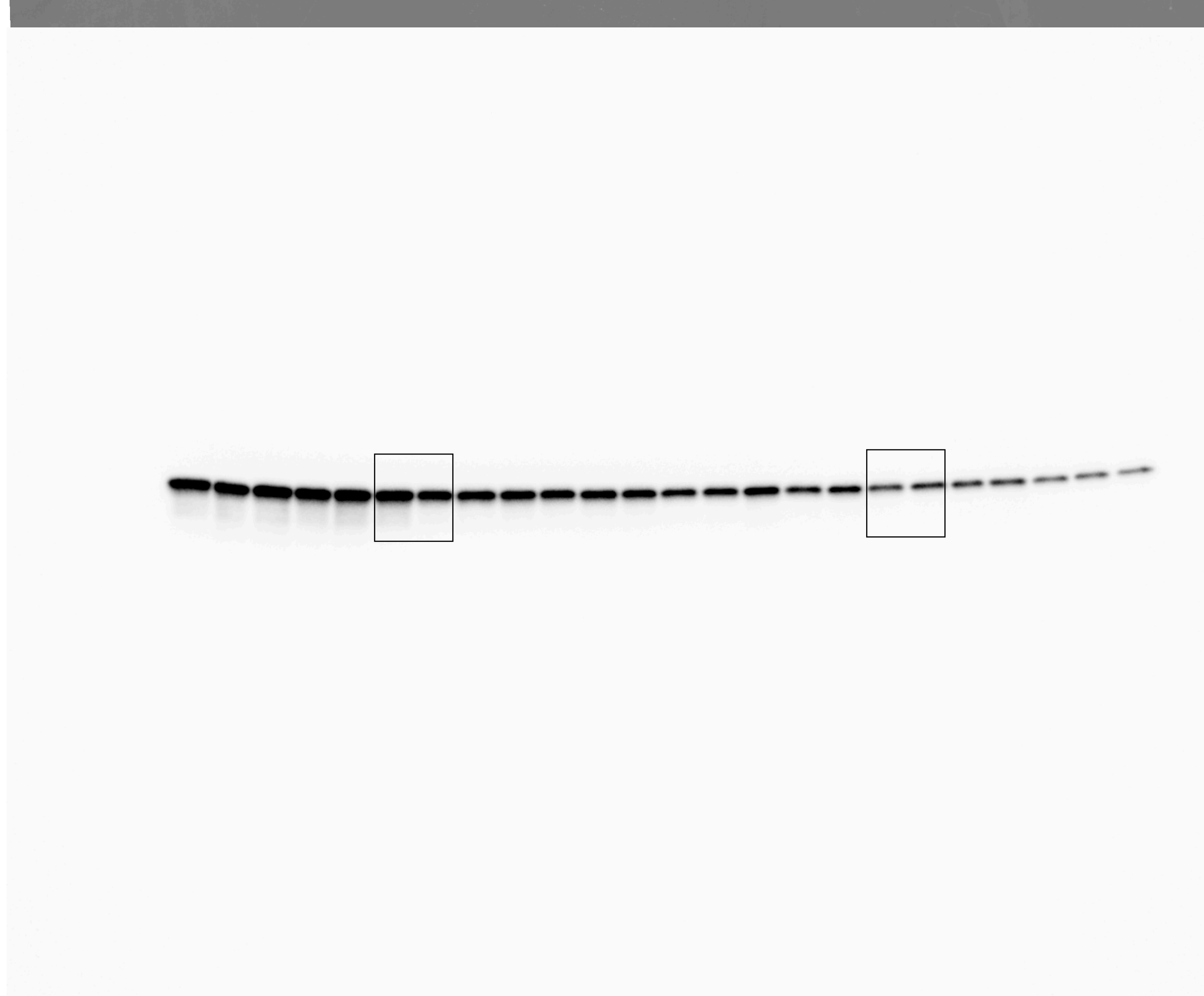

For co-IP experiment desitometric analysis

Experiment #1

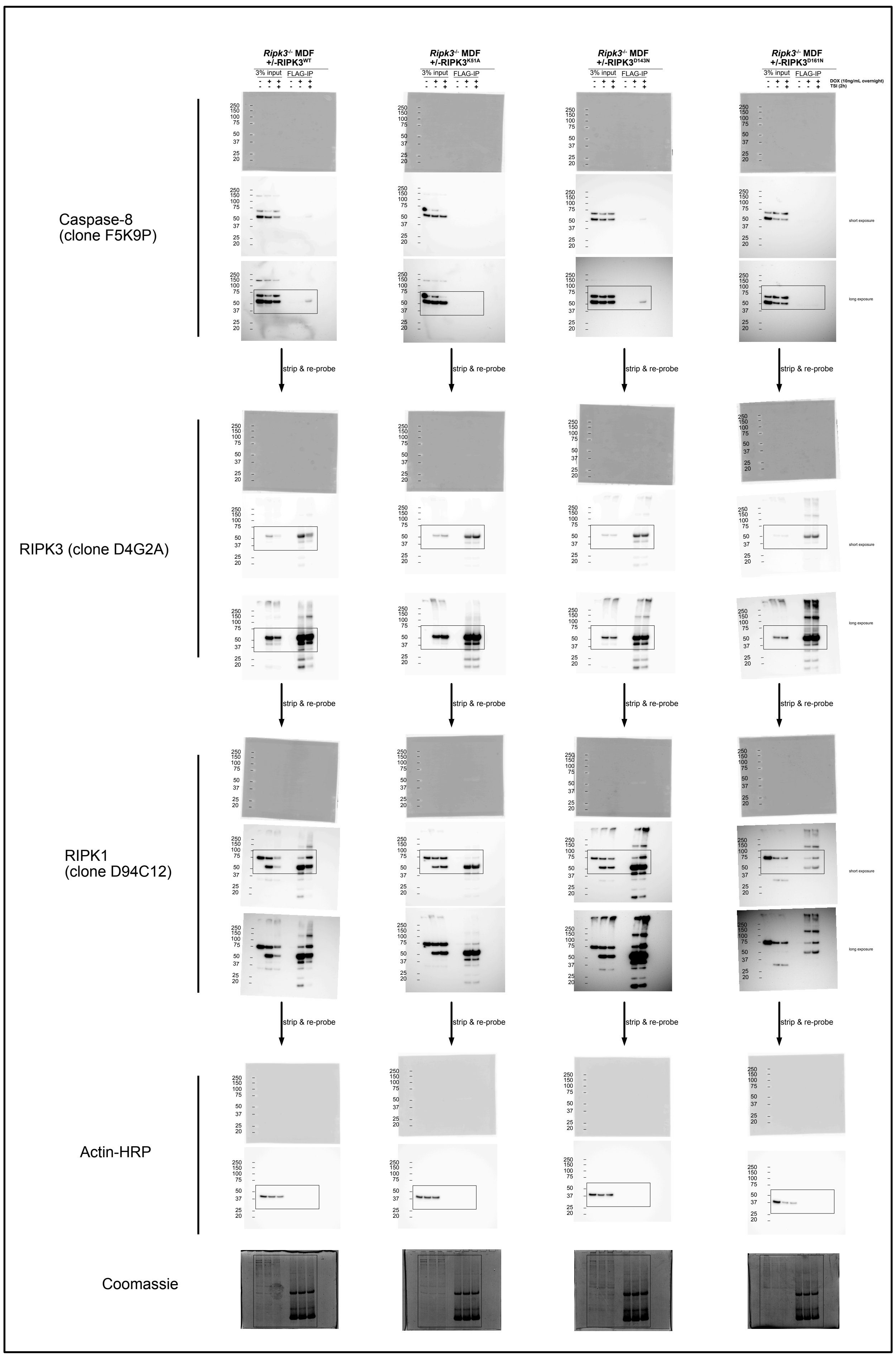

Experiment #2

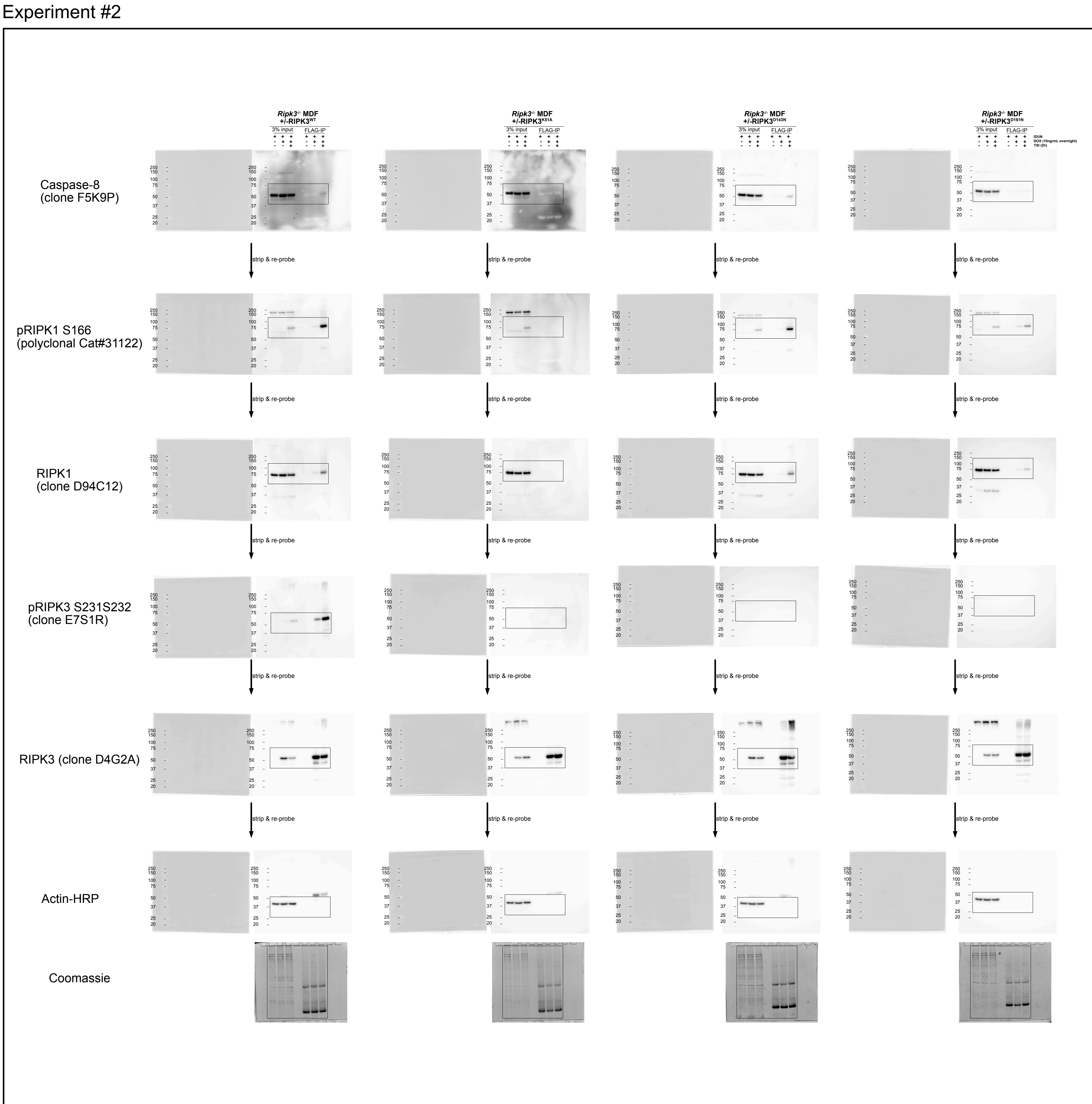

Supplement: Supplementary file 2 [file 41418_2026_1677_MOESM2_ESM.pdf]
